# Case Report on Deep Brain Stimulation Rescue After Suboptimal MR-Guided Focused Ultrasound Thalamotomy for Essential Tremor: A Tractography-Based Investigation

**DOI:** 10.3389/fnhum.2020.00191

**Published:** 2020-06-26

**Authors:** Sabir Saluja, Daniel A. N. Barbosa, Jonathon J. Parker, Yuhao Huang, Michael R. Jensen, Vyvian Ngo, Veronica E. Santini, Kim Butts Pauly, Pejman Ghanouni, Jennifer A. McNab, Casey H. Halpern

**Affiliations:** ^1^Department of Neurosurgery, Stanford University School of Medicine, Stanford, CA, United States; ^2^Department of Neurology and Neurological Sciences, Stanford University School of Medicine, Stanford, CA, United States; ^3^Department of Radiology, Stanford University School of Medicine, Stanford, CA, United States

**Keywords:** essential tremor, focused ultrasound, deep brain stimulation, tractography, thalamus, MRI

## Abstract

Essential tremor (ET) is the most prevalent movement disorder in adults, and can often be medically refractory, requiring surgical intervention. MRI-guided focused ultrasound (MRgFUS) is a less invasive procedure that uses ultrasonic waves to induce lesions in the ventralis intermedius nucleus (VIM) to treat refractory ET. As with all procedures for treating ET, optimal targeting during MRgFUS is essential for efficacy and durability. Various studies have reported cases of tremor recurrence following MRgFUS and long-term outcome data is limited to 3–4 years. We present a tractography-based investigation on a case of DBS rescue for medically refractory ET that was treated with MRgFUS that was interrupted due to the development of dysarthria during the procedure. After initial improvement, her hand tremor started to recur within 6 months after treatment, and bilateral DBS was performed targeting the VIM 24 months after MRgFUS. DBS induced long-term tremor control with monopolar stimulation. Diffusion MRI tractography was used to reconstruct the dentatorubrothalamic (DRTT) and corticothalmic (CTT) tracts being modulated by the procedures to understand the variability in efficacy between MRgFUS and DBS in treating ET in our patient. By comparing the MRgFUS lesion and DBS volume of activated tissue (VAT), we found that the MRgFUS lesion was located ventromedially to the VAT, and was less than 10% of the size of the VAT. While the lesion encompassed the same proportion of DRTT streamlines, it encompassed fewer CTT streamlines than the VAT. Our findings indicate the need for further investigation of targeting the CTT when using neuromodulatory procedures to treat refractory ET for more permanent tremor relief.

## Introduction

Essential tremor (ET) is the most prevalent movement disorder in adults. Treatment options for medically refractory cases include a variety of ablative and deep brain stimulation (DBS) procedures, usually targeting the ventralis intermedius (VIM) nucleus of the thalamus (Flora et al., 2010; Louis and Ferreira, 2010).

Recently, Elias et al. (2016) reported the results of a randomized control trial demonstrating the efficacy of unilateral MRI-guided focused ultrasound (MRgFUS) targeting the VIM in treating refractory ET. Out of the 56 patients who received MRgFUS thalamotomy of the VIM, 5 patients (8.9%) experienced the return of their tremor symptoms within 12 months postoperatively, with tremor scores worsening by 23% (Elias et al., 2016). In a 2-year follow up study, however, 4% of the original cohort subsequently received DBS due to unsuccessful or suboptimal treatment with MRgFUS (Chang et al., 2018). A retrospective comparative evaluation of RF thalamotomy, DBS, or MRgFUS for ET patients revealed this loss of effect is shared across modalities (Halpern et al., 2019). Moreover, compared to 6-months post-procedure, the 3-year follow-up study found that even though the primary outcome metric for the trial (i.e., the hand combined tremor-motor score) was significantly improved, there was a slight but significant increase in the median total Clinical Rating Scale for Tremor (CRST) score over time (Kim et al., 2017). The mechanism for this recrudescence remains elusive and is undoubtedly multifactorial, but a detailed review of the anatomic aspects of a suboptimal MRgFUS thalamotomy may guide the future management of these patients (Ravikumar et al., 2017).

One approach for understanding this loss of efficacy is utilizing diffusion-weighted MRI (dMRI) imaging to assess the white-matter fiber tracts being modulated by MRgFUS. Tractography studies have demonstrated that lesions must target the cerebello-thalamo-cortical network for treatment of ET (Coenen et al., 2014). The two major groups of white-matter fiber tracts involved in this network are the dentatorubrothalamic tract (DRTT) and the corticothalamic tract (CTT). These two pathways have been found to be necessary targets for the treatment of ET (Tian et al., 2018).

We present a tractography-based investigation of a patient treated with MRgFUS thalamotomy for ET, whose procedure was prematurely aborted due to new onset dysarthria. Immediately post-procedure, the patient experienced tremor relief and the dysarthria partially improved, but her tremor symptoms, most notably hand tremor, began to return 6 months postoperatively. The patient subsequently received DBS, and the surgery was well-tolerated and efficacious at the long-term.

Using a multimodal imaging strategy, we reconstructed the MRgFUS lesion and the volume of activated tissue (VAT) produced by the DBS electrode and the patient’s specific programming. We then used probabilistic tractography to assess the relationships between the MRgFUS lesion, DBS VAT, and the white matter fiber tracts associated with tremor control. This methodology offers a unique understanding of the specific fiber tracts modulated in both MRgFUS and DBS, in order to shed light on why DBS yielded a better long-term outcome in our patient.

## Case Description

A 70-year-old female with medically refractory ET was evaluated at our movement disorders clinic after nearly 30 years of tremor. Her tremor began in her left hand and eventually progressed to her right hand, head and voice. Eventually, she required assistance for her activities of daily living, including eating, writing, and dressing due to the severity of her tremor. She tried numerous medication therapies including combinations of propranolol, primodone, and gabapentin, in addition to chemodenervation with botulinum toxin. Despite all treatment attempts, she only achieved suboptimal tremor control.

**FIGURE 1:**
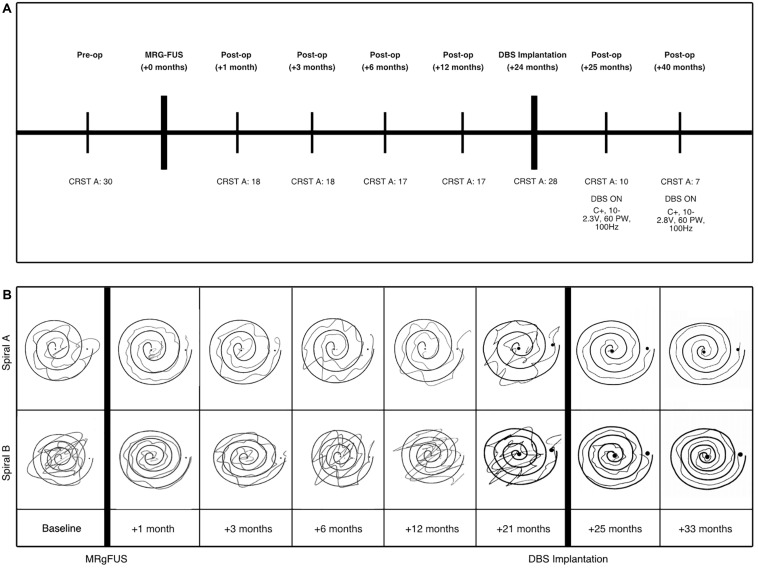
**(A)** Clinical timeline of patient’s procedures and DBS programming parameters following FUS and lead implantation. The patient’s tremor was evaluated using the Clinical Rating Scale for Tremor (CRST) A subsection, which evaluates tremor, including those of the upper extremities. **(B)** “Archimedes spiral” drawings (right hand only) during the B subsection of the CRST assessment to evaluate hand tremor at each time point to demonstrate tremor progression over time after MRgFUS procedure. Spiral A is wide, compared to the narrow spiral B (acquired as part of the CRST B).

At presentation, she was found to have postural tremors bilaterally in her upper extremities, significantly worsening with action and improving with rest. Her handwriting as well as her straight line and spiral drawing tests were markedly abnormal (Figure 1). She had head tremor and her voice was tremulous with audible oscillations. Her bedside cognitive status, as assessed by the Montreal Cognitive Assessment (MoCA) test, was within normal limits. There was no evidence of parkinsonism on examination. The CRST A subscore was 30 at the time of presentation in 2015, reflecting her postural and kinetic tremors (Figure 1). The patient presented with options of continued medical management, bilateral DBS, unilateral DBS, or MRgFUS as part of an ongoing clinical trial. At the time of initial presentation and evaluation, the patient was most distressed by her dominant hand tremor, and thus, after presented with the options, elected to proceed with MRgFUS focused on relief for her dominant upper extremity tremor.

The patient underwent a left MRgFUS thalamotomy in August 2015. The series of sonications is described in Supplementary Table S1. The left VIM nucleus target was guided by 3T MRI using standard coordinates from the mid-commissural point (MCP): –13.3 mm, –6 mm, 0 mm (∼10 mm from the ventricular wall) for sonications 1–19, 1 mm medial from the canonical stereotactic target. This target was chosen to provide about 2 mm of a safety margin from the thalamo-capsular boundary based on the patient’s preoperative MRI imaging. There were adjustments to the location of the sonication’s focus in order to sonicate the center of the planned target. By sonication 19, the patient’s tremor was largely relieved, but the lesion’s boundaries were approaching the internal capsule. For sonication 20, the target was moved 1 mm medially to avoid the internal capsule. For final sonication 21, the target was moved an additional 1 mm medial to continue the ablation but ensure no breach of the internal capsule. At the conclusion of sonication 21, transient dysarthria was noted on the patient’s clinical examination, and the procedure was terminated. Out of the 21 sonications performed, four of them reached a temperature greater than 55°C, and the maximum temperature attained was 61°C. The highest energy sonication reached 15940 J (797 W for 20 s). The SDR was 0.51. There were no cavitations. The procedure was aborted due to new-onset dysarthria. The patient experienced a significant improvement in her tremor at 2-week follow-up. Her only new symptom was transient dysarthria that initiated during MRgFUS treatment. Over the next 6 months, however, she noticed progressive tremor recurrence and worsening of her tremor symptoms despite partial improvement of dysarthria (see Supplementary Table S2).

After discussion with and further evaluation of the patient through a multidisciplinary DBS review board, she was deemed a candidate for DBS targeted to the VIM nucleus. Approximately 24 months after her initial left MRgFUS, DBS (Medtronic Activa PC) leads were bilaterally placed without complication using frameless robotic-assisted stereotactic navigation (Ho et al., 2019). Based on the dysarthria previously experienced that was presumed to be due to the relatively medial location of the MRgFUS treatment, DBS leads were placed using target coordinates of -13, -6.5, and 0 mm from the MCP (5.9 mm anterior to PC). The target was more lateral in order to minimize dysarthria, and more posterior so that the trajectory did not enter blood vessels, ventricles, or sulci. Intraoperative electrophysiological monitoring and postoperative imaging demonstrated satisfactory lead placement. A standard monopolar testing protocol was performed to evaluate the threshold of efficacy for each contact and any adverse effects. With stimulation at 1.5 V, there were no adverse effects with activation of contacts C1-C3, however, the patient experienced transient right lip paresthesia with activation of left hemisphere contact C0, which was the contact located closest to the MRgFUS lesion. At 3–4 V, the patient experienced slight dysarthria when left hemisphere contact C1 was activated. When C1 in the right hemisphere was activated at 3.0 V, her dysarthria worsened. Contact C2 was chosen for monopolar activation at 2.7 V in the left hemisphere and 2.0 V in the right hemisphere, which maximized her tremor suppression and minimized adverse effects. At her last evaluation (16-month follow-up after DBS), she had consistent and effective tremor control, with a CRST score of 7 (Figure 1). The left lead was active at contact 2 set at 2.8 V, pulse width 60 ms and 100 Hz. The right lead was active at electrode 10 set at 2.3 V, pulse width 60 ms and 100 Hz. The patient had excellent tremor suppression following monopolar activation of the DBS leads, as shown by sustained decrease in CRST and significant improvement drawing coherent spirals.

## Methods

### MRI Imaging Acquisition and Preprocessing

T1-weighted and T2-weighted structural 3T MRI images were acquired before and after FUS. Diffusion-weighted images were acquired from the patient before MRgFUS (3T, 2 mm isotropic, TR/TE = 8500/81.6 ms, b = 2500 s/mm^2^, 60 directions, 582 s) and before DBS implantation (3T, 2 mm isotropic, TR/TE = 8000/60.7 ms, b = 1000 s/mm^2^, 30 directions, 502 s). Computed tomography (CT) images with 1 mm slice thickness were obtained postoperatively after DBS implantation. FSL’s “topup” tool was used to estimate and correct non-zero off-resonance fields caused by susceptibility distribution of the subject’s head via analysis of forward and reverse phase encoded B_0_ image acquisitions (Andersson et al., 2003). FSL’s “eddy” tool was used to correct for the eddy current caused by rapid switching on and off of the diffusion gradient (Smith et al., 2004).

### Lesion Volume, Electrode Reconstruction, and Volume of Activated Tissue Estimation

MRgFUS results in three distinct zones of ablation that can be viewed on a T2-weighted image (Wintermark et al., 2014). The lesion region of interest (ROI) was created by including the voxels that are within the two inner zones (the third outer zone being vasogenic edema) on an MRI acquired the same day following MRgFUS thalamotomy. Lead-DBS was used for localization and visualization of the DBS electrode contacts (Horn et al., 2019). Linear and nonlinear transformations were computed from the MNI 152 2009c template to the T1 and T2-weighted images, as well as the postoperative CT. The DBS Intrinsic Template Atlas (DISTAL) was subsequently transformed onto the native T1-weighted images and used to localize the electrodes as well as the MRgFUS lesion in reference to the VIM (Ewert et al., 2018). The VAT was estimated using a finite element modeling method based on the characteristics of the brain tissue activated and the DBS programming voltage and estimated impedance (Madler and Coenen, 2012).

### Probabilistic Tractography and Statistical Analysis

Tractography was performed with MRtrix using constrained spherical deconvolution to estimate the white-matter fiber orientation distribution from the diffusion signal of the dMRI images (Smith et al., 2013). Using probabilistic tractography, the DRTT was filtered to include white-matter tracts that are seeded at the dentate nucleus and terminate in the thalamus, along with sending collaterals to the red nucleus. Freesurfer was used to segment the structural T1-weighted images to generate ROIs for the thalamus, and dentate nucleus (Desikan et al., 2006; Fischl, 2012). The red nucleus was drawn using guidance from an expert neuroradiologist. The CTT was filtered to include only white-matter tracts seeded at the precentral gyrus and terminate at the thalamus. Freesurfer was used to generate masks encompassing the precentral gyrus.

A mask of the MRgFUS lesion was overlaid on the pre-MRgFUS tractography streamlines, and a mask of the VAT was overlaid onto the pre-DBS streamlines. The proportion of streamlines of each tract that were incorporated by the lesion and VAT were calculated by dividing the raw number of streamlines of the DRTT and CTT that intersected the lesion and VAT, by the total number of streamlines within the DRTT and CTT.

## Diagnostic Assessment and Results

The MRgFUS lesioning procedure in the VIM resulted in immediate tremor suppression. The patient’s CRST A score decreased from 30 to 18 as a result. Her tremor suppression remained stable for 6 months, then began worsening. Twenty-four months after the MRgFUS procedure, the patient’s CRST A score had increased to 28, and at this time, the patient received DBS electrode implantation. Subsequent programming reduced her tremor, resulting in a CRST A score of 7 after optimizing DBS programming parameters.

**FIGURE 2:**
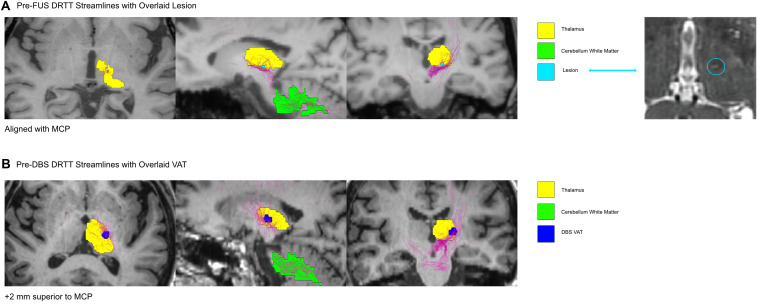
The DRTT shown in the Pre-FUS and Post-FUS volumes. In **(A)**, the lesion after MRgFUS was overlaid onto the pre-FUS image, to isolate the voxels that encompassed the lesion before the MRgFUS procedure. In **(B)**, the DBS VAT was overlaid onto the Pre-DBS image. The DRTT was isolated from all tracts generated via probabilistic tractography by only including the streamlines that intersected the ROI masks for the cerebellum white matter (dentate nucleus), thalamus, and red nucleus.

**FIGURE 3:**
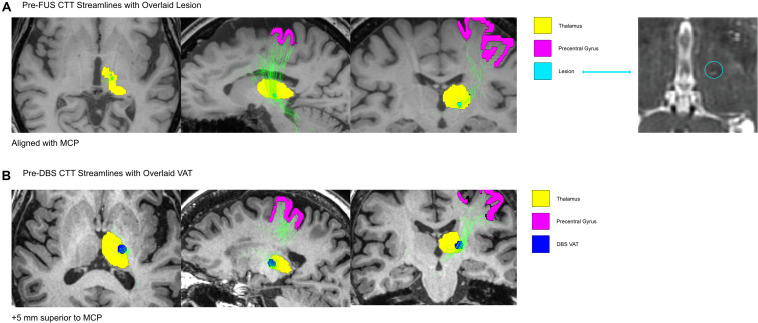
The CTT shown in the Pre-FUS and Post-FUS volumes. In **(A)**, the lesion after MRgFUS was overlaid onto the pre-FUS image, to isolate the voxels that encompassed the lesion before the MRgFUS procedure. In **(B)**, the DBS VAT was overlaid onto the Pre-DBS image. The CTT was isolated from all tracts generated via probabilistic tractography by only including the streamlines that intersected the ROI masks for the precentral gyrus and thalamus.

Probabilistic tractography reconstructed streamlines of the DRTT (Figure 2) and CTT (Figure 3) for the pre-FUS (A) and pre-DBS (B) diffusion weighted images. The lesion after MRgFUS, and the VAT from DBS, were overlaid onto each respective image to select the voxels that were modulated by each modality.

The MRgFUS lesion’s volume was calculated to be 20.28 mm^3^. The estimated VAT from the unilateral left VIM DBS at the patient’s last programming settings was 233.16 mm^3^. The VAT’s x, y, and z coordinates relative to the MCP were -15.5, -2.5, and -7 mm. The center of the lesion was located 3.75 mm closer to the midline than the active DBS VAT, and 5 mm more ventral. The lesion location in the pre-FUS image captured 12.9% of the DRTT streamlines and 4.4% of the CTT streamlines, while the DBS VAT location of the pre-DBS image encompassed 13.6% of the DRTT streamlines and 29.7% of the CTT streamlines, respectively.

**FIGURE 4:**
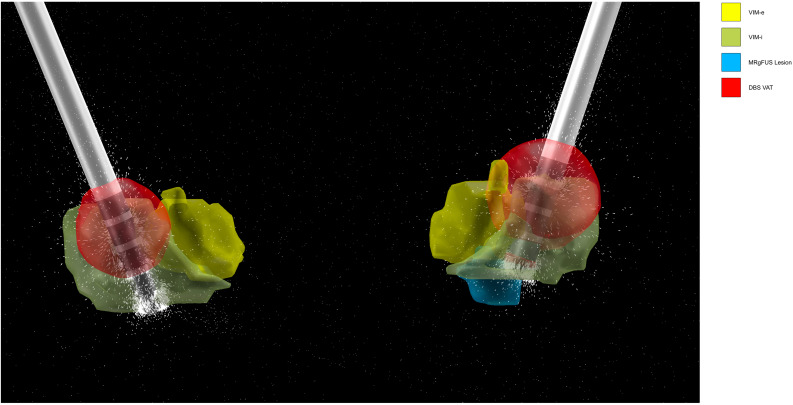
Bilateral DBS (left hemisphere on right side, right hemisphere on left side) 3D lead reconstruction and VAT generation in LEAD-DBS. DBS electrode contact spacing models design of Medtronic 3389. The dashes represent the directionality of the induced electric field from the activated contact, in this case contact 2. The lesion and VAT are localized alongside the internal and external segments of the VIM (VIM-i and VIM-e, respectively) defined by the DISTAL atlas.

For visualization purposes, the lesion and VAT were reconstructed in 3D alongside the DBS electrodes and the internal and external nuclei of the VIM, defined by the DISTAL atlas (Figure 4).

## Discussion

Tremor relief that is not sustained after MRgFUS treatment is troublesome to patients and presents a significant management challenge. For these patients in whom treatment has failed, it may be appropriate to offer repeat or rescue procedures (Tuleasca et al., 2017; Wang et al., 2018; Weidman et al., 2019). However, in the described case, the scant availability of repeat MRgFUS efficacy, the side effect of dysarthria, and patient preference for bilateral therapy made DBS a favorable alterative, not to mention its ability to be used somewhat reversibly and bilaterally. Using patient-specific probabilistic tractography, we investigated this case of medically refractory ET treated with MRgFUS and subsequently DBS to retrospectively evaluate the topography and fiber tracts modulated in both procedures in order to understand their differential efficacy and side-effect profile. Importantly, the MRgFUS lesion in this case was not optimized due to aborting the procedure. However, we feel optimizing targeting based on reports such as this may prevent future MRgFUS treatment failures.

Our technique of comparing the overlap in the lesion/VAT module volume with patient-specific tracts suggested the difference in treatment outcomes may be explained in part by the DBS VAT. The MRgFUS lesion was located ventromedially to the VAT (Figure 4), and was also significantly smaller, comprising roughly 10% of the volume of the VAT. The MRgFUS lesion was placed medially to avoid heat extending into the internal capsule, and thus sonications were moved serially more medially as the procedure continued to avoid heating of the pyramidal tract. DBS electrodes were placed in a similar trajectory, with the most distal contact (contact 0) bordering the lesion location. During programming, the patient received the most tremor suppression when contact 2 was activated, moving the VAT dorsolaterally.

Although the tractography findings suggest that more accurate targeting and larger VAT resulted in more sustained tremor relief, our case also adds to a further body of evidence about the variable efficacy of MRgFUS thalamotomy. It is noteworthy that despite suboptimal targeting and premature cessation of sonications due to dysarthria, the initial MRgFUS treatment resulted in tremor relief, albeit temporarily. The onset of tremor recurrence within 1 year postoperatively has been reported in numerous cases in the literature, even without lesions complicated by dysarthria (Wang et al., 2018; Halpern et al., 2019). This suggests several plausible explanations for loss of tremor suppression efficacy over time after MRgFUS. First, the lesion created may have a penumbra region of edemanous brain where reversible neuromodulatory rather than neuroablative effects predominate. Additionally, ongoing pathologic remodeling occurring among Purkinje and other cell types in the cerebello-thalamic tremor circuit may lead to progressive worsening of tremor (Louis and Faust, 2020) in the face of a well-targeted MRgFUS lesion. Further investigation should be conducted on the time course of cellular mechanisms of thalamic MRgFUS lesions in tremor model systems as well as neuroimaging studies to uncover predictive imaging biomarkers for tremor recurrence.

Additionally, our tractography analysis here investigates a single patient’s structural connectivity, but insight can be drawn into the differences in streamline counts within the DRTT and CTT, which have been reported to be necessary when using tractography to define patient-specific neuromodulatory targets for ET (Coenen et al., 2014). The negligible difference in the proportion of DRTT streamlines modulated by MRgFUS and DBS indicates that both modalities targeted this tract in a similar way, although a different location within the DRTT was modulated in each procedure. However, when comparing DBS stimulation to MRgFUS, we found a large increase in the proportion of CTT streamlines residing within the DBS VAT, compared to those found within the patient’s MRgFUS lesion volume. Although prior work has shown that disruption of cerebellar input into the ventral thalamus is necessary to disrupt tremor pathophysiology (Gallay et al., 2016), the pattern suggests that targeting the DRTT alone may not be sufficient, thus future investigations should explore the role of modulating the CTT to maintain clinical effectiveness of tremor relief and balancing the use of imaging to guide targets with intra-procedural findings. This is in line with findings by Tian et al., which report that the most efficacious target, in a cohort of ET patients who received MRgFUS thalamotomy, encompassed both the CTT and DRTT (Coenen et al., 2014).

Our findings demonstrate modeling white-matter fiber tracts using probabilistic tractography may serve as a method to inform and optimize targeting of initial MRgFUS lesions and tailor rescue procedures for those with recurrent or persistent tremor. We have demonstrated that the larger size of the DBS VAT, compared to the MRgFUS lesion, incorporates a larger area of white-matter to be targeted, allowing for the inclusion of more fibers of the CTT, as it has been reported that the size of the lesion is positively correlated to improved treatment outcome (Federau et al., 2018).

Dysarthria is a common adverse effect of neuromodulatory procedures targeting the VIM, including both MRgFUS and DBS. While every attempt is made to mitigate this effect across procedural modalities, indirect targeting of VIM lends itself to suboptimal accuracy of sonications and DBS lead placement. This effect may be caused by stimulation or sonications of the posterior limb of the internal capsule. Activation of ventral contacts in the VIM have also shown to stimulate the homuncular representation of the head (Montgomery, 2010). Moreover, the patient’s absence of dysarthria after successful DBS treatment suggested the dysarthria was due to medial sonications as the DBS lead was relatively lateral. Using tractography to optimize targeting is one approach that we highlight in this case study to attempt to mitigate such troublesome adverse effects. We believe our case underscores the importance of tractography-based targeting, which becomes particularly relevant given that VIM targeting is indirect due to our inability to segment thalamic nuclei via conventional MRI (Coenen et al., 2014). There have been reported attempts to directly modulate white-matter tracts via DBS, such as the DRTT through targeting the posterior subthalamic area (PSA), which appear to be effective at suppressing tremor (Dembek et al., 2020). The findings of our case report further support the idea that indirect VIM targeting may not be sufficient alone to optimize outcomes for ET (Benabid et al., 1991). Tractography utilizes the diffusion signal of the white matter tracts in the brain, which is personalized to each patient and more directly tells us where to target. In particular, the canonically activated contacts for VIM DBS are usually ventrally located (Gallay et al., 2016), but the most effective contact in our patient was the more superior contact 2. Moreover, Boutet et al. (2018) have shown that medially placed lesions in the VIM were associated with 41 times the likelihood of speech adverse effects. Our case report supports these findings.

A limitation of this work comes from the relatively low spatial resolution of dMRI at roughly 2 mm isotropic. This indicates that tractography-based targeting should be used alongside other targeting methods, such as atlas coregistration, intraoperative microelectrode recordings, and/or real-time patient examination, to ensure accurate tract localization. Another limitation includes that our dMRI acquisitions taken pre-MRgFUS and pre-DBS had different acquisition parameters. We have accounted for this difference by comparing the proportion of streamlines targeted by each method, rather than the raw streamline count, which may be more affected by varying acquisition parameters. It is also important to note that in this case, the DRTT was within the lesional zone of the MRgFUS, and the CTT was additionally included within the DBS VAT; further investigation should be conducted to determine the effects of including CTT targeting by MRgFUS. The findings of this report highlight the need for prospective validation of tractography-based targeting and modeling of modulation by lesioning and electrical stimulation modalities such as DBS.

## Data Availability Statement

The datasets generated for this study are available on request to the corresponding author.

## Ethics Statement

Written informed consent was obtained from the patient for the publication of this case report.

## Author Contributions

SS was the main author on this case report. DB, JP, YH, MJ, and VN helped with data acquisition, analysis, and editing. VS was the neurologist involved with clinical evaluation and care of the patient. PG, VS, KP, and JM were involved in the clinical trial for MRI-guided Focused Ultrasound that our patient was enrolled in and assisted with the methodologies for data acquisition and analysis. CH was the neurosurgeon in the clinical trial involved in the localization of the lesions and assessments post-procedure.

## Conflict of Interest

PG, VS, and CH did receive percent effort support from Insightec during the pivotal trial, in which this patient was included. The remaining authors declare that the research was conducted in the absence of any commercial or financial relationships that could be construed as a potential conflict of interest.
