# Supplementary material for: Case Report on Deep Brain Stimulation Rescue After Suboptimal MR-Guided Focused Ultrasound Thalamotomy for Essential Tremor: A Tractography-Based Investigation
Source: Front Hum Neurosci. 2020 Jun 26;14:191. doi: 10.3389/fnhum.2020.00191 (PMC7333679; doi:10.3389/fnhum.2020.00191)
Supplement: Supplementary file 1 [file Table_1.DOCX]

| **MRgFUS Sonication Treatment Timeline** | | |
| --- | --- | --- |
| **Sonication** | **Movement** | **Rationale / Observation** |
| 2 | 0.4 mm Medial (Alignment) | Heating observed lateral to target |
| 8 | 0.8 mm Inferior (Alignment) | Heating observed superior to target |
| 10 | 0.6 mm Inferior (Alignment) | Heating observed superior to target |
| 17 | No Movement | Improvement in Tremor |
| 19 | 1.0 mm Medial (Target) | Lesion 7mm diameter, encroaching on internal capsule |
| 20 | 1.0 mm Medial (Target) | Moved to target head/voice somatotopy within VIM |
| 21 | No Movement | Dysarthria noted. Postural and rest tremor relieved, small residual intention tremor. |
| *Note: For sonication numbers not listed, no changes were made to the targeting of MRgFUS array. Alignment adjustments as noted, were made to achieve accurate targeting of tissue heating and do not represent movement of the intended anatomical lesion target. Movements made after sonication 19 represent adjustments of the anatomic region (target) of tissue heating for clinical purposes.* | | |
