# Supplementary material for: Case Report on Deep Brain Stimulation Rescue After Suboptimal MR-Guided Focused Ultrasound Thalamotomy for Essential Tremor: A Tractography-Based Investigation
Source: Front Hum Neurosci. 2020 Jun 26;14:191. doi: 10.3389/fnhum.2020.00191 (PMC7333679; doi:10.3389/fnhum.2020.00191)
Supplement: Supplementary file 2 [file Table_2.DOCX]

**Supplementary Table 2– CRST Scores Over Time**

| Time point | CRST A | CRST B | CRST C | CRST Total |
| --- | --- | --- | --- | --- |
| Screening | 24 | 28 | 17 | 69 |
| Baseline | 30 | 29 | 19 | 78 |
| +1 month | 18 | 19 | 5 | 42 |
| +3 months | 18 | 24 | 12 | 54 |
| +6 months | 17 | 22 | 12 | 51 |
| +12 months | 18 | 29 | 17 | 64 |
| +24 months (DBS Implantation) | 28 | -- | -- | -- |
| +25 months | 10 | -- | -- | -- |
| +40 months | 7 | -- | -- | -- |
